# Allergic reaction to platelet-rich plasma (PRP): Case report

**DOI:** 10.1097/MD.0000000000014702

**Published:** 2019-03-08

**Authors:** Michal Latalski, Alicja Walczyk, Marek Fatyga, Erich Rutz, Tomasz Szponder, Tomasz Bielecki, Anna Danielewicz

**Affiliations:** a Department of Paediatric Orthopaedics, Medical University of Lublin; b Clinic of Paediatric Allergology, University Children Hospital, Lublin, Poland; c Department of Pediatric Orthopedic, University Children's Hospital, UKBB, Basel, Switzerland; d Department and Clinic of Animal Surgery, Faculty of Veterinary Medicine,University of Life Sciences in Lublin; e Department and Clinic of Orthopaedics, Trauma and Reconstructive Surgery, Trauma Center St. Barbara Hospital, Medical University of Silesia, Sosnowiec, Poland.

**Keywords:** augmentation, bone cyst, bone lesion, PRP, side effect

## Abstract

**Rationale:**

In the recent years, growing interest is focused on the use of platelet-rich plasma (PRP) in wound healing and tissue regeneration. There are a number of papers regarding the usefulness of PRP in the healing of ulcerations, skin injures, bone loss or distraction osteogenesis. Most authors emphasize the safety of PRP usage due to its authogenic nature.

**Patient concerns:**

We present a case of a 14 -year-old boy admitted to our department due to simple bone cyst of the distal tibia, qualified for injection of PRP into the cyst. PRP was separated with the use of Magellan Autologous Platelet Separator System (Arteriocyte Medical Systems Hopkington, MA) according to the manufacturers’ manual. Immediately after separation during short-term IV anaesthesia, 3 mL of PRP was installed to the bone cyst under image intensifier control.

**Diagnoses:**

Within the first 24 hours after exposure to PRP, the skin rash appeared. Physical examination revealed the small red papular, regionally purpuric eruptions, mainly concentrated on the upper extremities and on more warmed regions of skin, in association with pharyngitis, tonsillar enlargement, mucopurulent discharge in the posterior pharynx and swelling of the eyelids.

**Interventions:**

As the patient received calcium citrate with the PRP injection additional calcium citrate test were performed. Skin prick testing (negative) was and an intradermal test was positive (10×13 mm). Treatment included Claritine (Loratidinum) and Clemastin (Clemastinum)—both antihistaminic drugs.

**Outcomes:**

All symptoms withdrew and the patient was released home after 4 days. The patient is in 6 years follow-up without any symptoms of allergic disease.

**Lessons:**

Our case shows that safety of use of PRP is not absolutely sure. The pure autologous tissue is safe, but preparation for its use can substantially decrease this safety. In our patient, only limited skin reaction to calcium citrate was observed, but general reaction leading to anaphylactic shock cannot be excluded. In order to reduce the risk of side effects skin test should be performed but as there were no records of allergic diseases on family and patients medical history this should apply to all patients.

## 1 Introduction

In the recent years, growing interest is focused on the use of platelet-rich plasma (PRP) in wound healing and tissue regeneration. There is a number of papers regarding the usefulness of PRP in the healing of ulcerations, skin injures, bone loss, symptomatic partial rotator cuff tears or distraction osteogenesis.^[1–4]^ Evidence of its efficacy has been mixed and highly dependent on composition and on the specific indication.^[5–7]^ Most authors emphasize that the PRP is a promising treatment modality with clear evidence of safety due to its authogenic nature.^[8]^ However the safety should be cast to doubt. The aim of this article is to present a case with an allergic reaction to platelet-rich plasma.

## 2 A case

**Figure 1:**
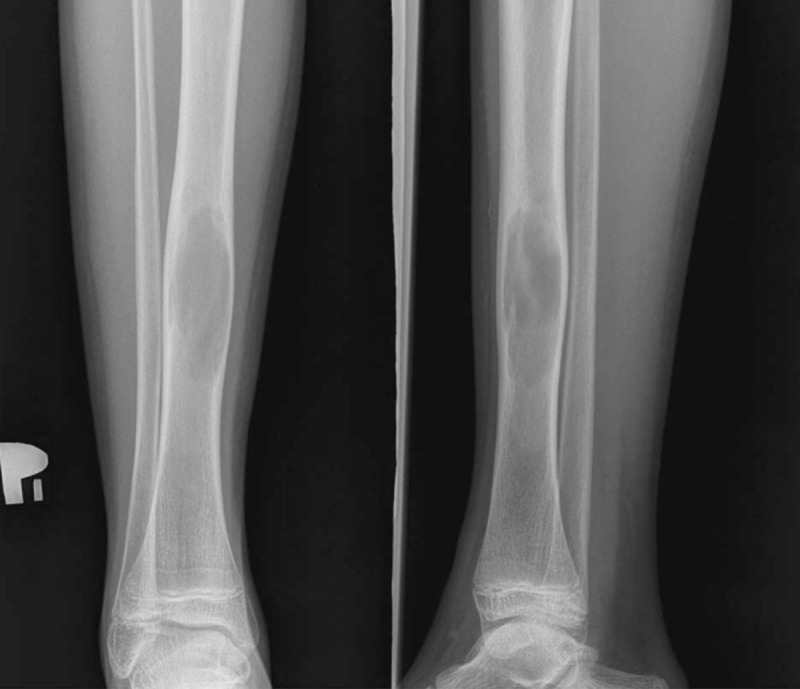
AP radiograph of the simple bone cyst of a distal/proximal tibia.

We present a case of a 14 -year-old boy admitted to our department due to simple bone cyst of the distal tibia (Fig. 1). Medical University review board approved the study (ethical board approval number 419/2013) and informed written consent was obtained from the patient for publication of this case report and accompanying images.

The patient was qualified for injection of PRP into the cyst.^[9]^ Original blood test results are presented in Table 1. The procedure has been conducted in the operation room. PRP was separated with the use of Magellan Autologous Platelet Separator System (Arteriocyte Medical Systems Hopkington, MA) according to the manufacturers manual. The Magellan kit consists of an automated centrifuge, a sterile container, 3 syringes with capacities of 60, 10, and 5 mL, and separating chambers with connectors. The set is completed by a vial with 30 mL of anticoagulant citrate dextrose solution A (ACD-A). The technique to obtain PRP is to place separation chambers in the centrifuge and connect them with a syringe containing 56 mL of blood obtained from a patient mixed with 4 mL of anticoagulant. The microprocessor-controlled centrifuge automatically divides the blood sample into these fractions. Three milliliters of PRP are transferred into a 10-ml syringe, which is then ready for use. Immediately after separation during short-term iv anaesthesia (Propofol), 3 mL of PRP was installed to the bone cyst under image intensifier control. Immediately after the injection the patient was a-symptomatic regarding blood pressure, heart rate, and local reaction at the side of injection.

**Table 1:**
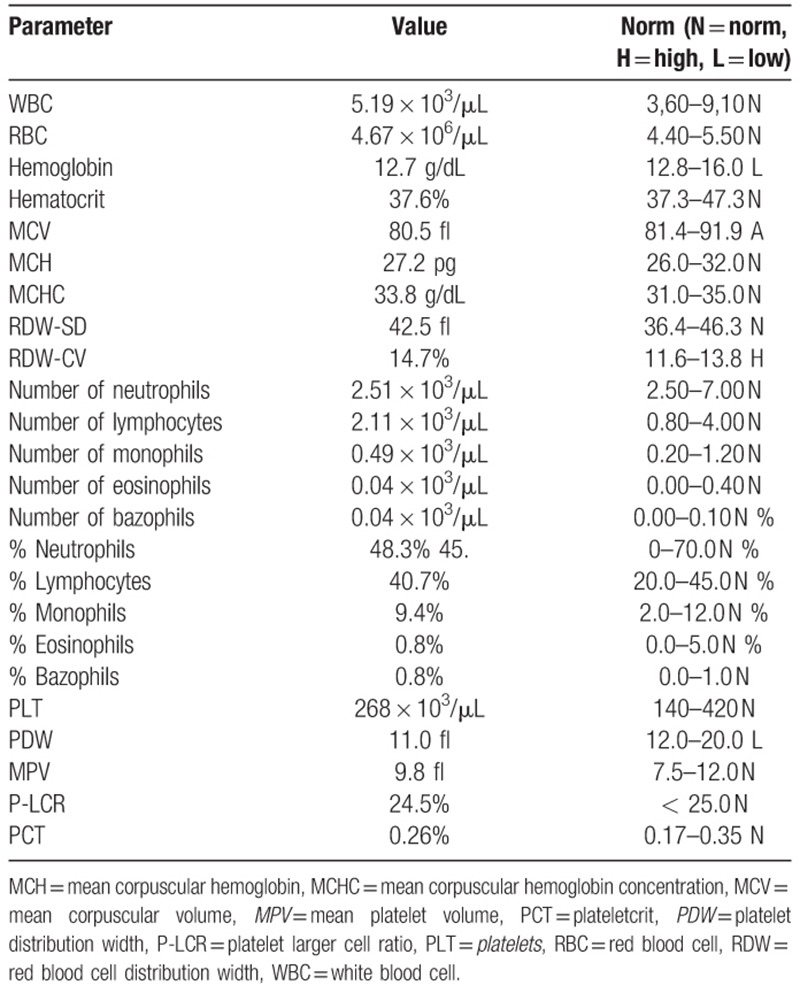
Original blood test results.

Within the first 24 hours after exposure to PRP, the skin rash appeared. Physical examination revealed the small red papular, regionally purpuric eruptions, mainly concentrated on the upper extremities and on more warmed regions of skin, in association with pharyngitis, tonsillar enlargement and mucopurulent discharge in the posterior pharynx.

Patient has been transferred to the Department of Allergology, admitted in generally good condition, temperature within normal range and heart rate 78/min. Additionally to the above-mentioned symptoms, swelling of the eyelids was found. On laboratory findings, acute phase reactants were normal (Table 2). Total immunoglobulin IgE was 171kU/l

**Table 2:**
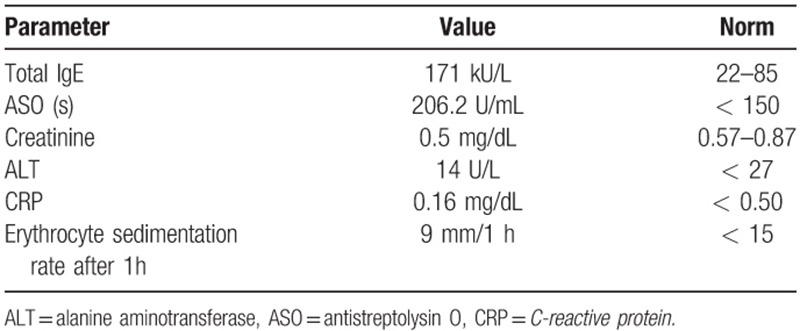
Blood test results after PRP injection.

Serum levels of IgE specific for food and inhaled allergens, both skin prick testing and intradermal testing were negative. (Allergology panel Paediatric kit is presented in Table 3). Intradermal testing using autologous serum was negative. There were no records of allergic diseases in family and patients medical history.

**Table 3:**
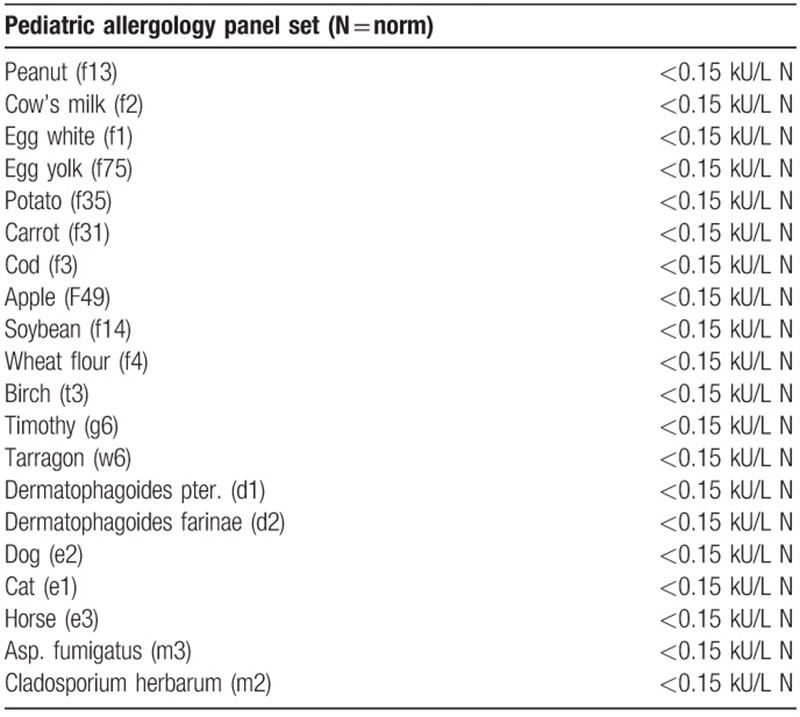
Patient's allergology panel pediatric kit.

As the patient received calcium citrate with the PRP injection additional calcium citrate test were performed. Skin prick testing (negative) was and an intradermal test was positive (10×13 mm). Treatment included Claritine (Loratidinum) and Clemastin (Clemastinum)—both antihistaminic drugs without PRP removal from the bone cyst. Symptoms alleviated and the patient was discharged home after 4 days. Ten months later he was admitted to the Allergology Department for control tests. Results are presented in Table 4. The patient is in 6 years follow-up without any symptoms of allergic disease.

**Table 4:**
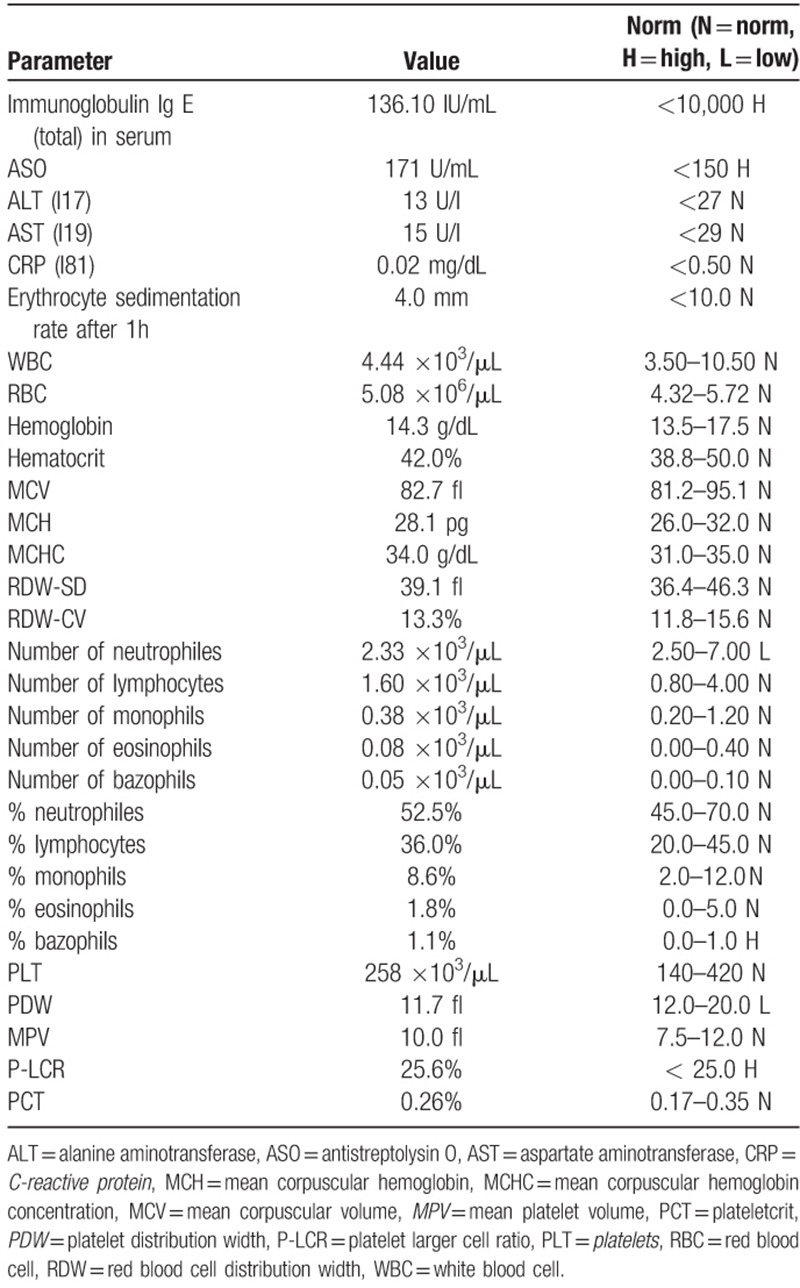
Blood test results after 10 months.

## 3 Discussion

The usage PRP in medicine is very common. The number of randomized controlled trials proving effectiveness is growing. Some authors reported treatment-related adverse events, but number of papers describing adverse side effects of PRP treatment are very limited.^[10–20]^ Bielecki et al described a side-effect induced by the combination of a demineralized freeze-dried bone allograft and leucocyte and platelet-rich plasma (L-PRP) during treatment for large bone cysts.^[21]^ He presented in 4-year follow up clinical study the influence of L-PRP gel on healing large solitary bone cysts in 6 patients. Although recent investigations have confirmed the osteo-inductive properties of L-PRP gel in vitro^[21]^ he showed that mixing allografts and L-PRP gel in the treatment of large cystic lesions is not efficient and it might induce unknown local reactions between them causing complete bone graft destruction.

Driver et al^[24]^ with a multicenter clinical study evaluated the safety and efficacy of autologous platelet-rich plasma gel for the treatment of nonhealing diabetic foot ulcers in 129 cases. The study showed an increase in the blood urea nitrogen in the control group, and an increase in either the thrombin time or the activated partial thromboplastin time was observed in both treatment groups (PRP and control). Senet et al^[25]^ in the trial on the local biological effect of autologous platelets used as adjuvant therapy for chronic venous leg ulcers reported 2 patients with dermatitis (one in each treatment group), one patient developed an infection in an existing ulcer, and one had thrombophlebitis (both in the PRP group). Overall, all studies agreed that there were not treatment-related complications per se.

In our case the allergy reaction appeared right after the PRP injection.

Despite negative results of skin tests with for food and inhaled allergens one could suspect type I hypersensitivity reaction due to the time of reaction, elevated serum levels of IgE and positive result of the intradermal test with calcium citrate. In this case, calcium citrate should be accounted as haptene. Type I hypersensitivity reaction is a result of stimulation by allergen IgE sensitized mast cell. Mast cells release mediators that cause inflammatory reactions typical for Type I hypersensitivity. It is also known that mast cells release a number of cytokines.^[26,27]^

There are many systems for obtaining platelet-rich plasma. Many publications comparing those systems in terms of plasma volume, white blood cell, red blood cell count, and growth factor concentrations like platelet-derived growth factors, transforming growth factor beta-1 (TGF-β1), and vascular endothelial growth factor (VEGF) were written. Although there is a lack of data about anticoagulants used in those preparation kits and their influence on the plasma sample.^[5–11]^

Most popular anticoagulants which are applied are heparin, citrate, acid citrate dextrose (ACD) and citrate-theophylline-adenosine-dipyridamole (CTAD). Heparin is a highly sulfated glycosaminoglycan polyanion that was first isolated from the bovine liver in 1916, is widely used as an injectable anticoagulant. It is a natural factor in preventing the clotting of blood in the blood vessels, acting as a brake on all phases, mainly in the phase of transition of prothrombin to thrombin and its effect on fibrinogen. Heparin activates antithrombin—the plasma-derived factor that inhibits the action of thrombin. Heparin is efficient and instantaneous in its anticoagulation, quite safe and cheap so that heparin can be administered with ease to patients.^[26,27]^ Citrate (sodium citrate) was first reported as an anticoagulant for hemodialysis in the 1960s by Morita et al and as an alternative regional anticoagulation in patients ongoing CRRT (continuous renal replacement therapy) in 1990 by Metha et al. Since then, citrate has gained more and more popularity. Citrate provides a regional anticoagulation virtually restricted to the extracorporeal circuit, where it acts by chelating ionized calcium. Citrate anticoagulation does not increase patient risk of bleeding like heparin. Acid citrate dextrose is a solution of citric acid, sodium citrate and dextrose in water. It is mainly used as an anticoagulant to preserve blood specimens required for tissue typing, it is also used during procedures such as plasmapheresis instead of heparin. There are 2 types ACD solutions, solution A and B. ACD is used as an anticoagulant in the extracorporeal blood processing with autologous PRP systems in the production of PRP. Citrate-based anticoagulants prevent the coagulation of blood by virtue of the citrate ion's ability to chelate ionized calcium present in the blood to form a non-ionized calcium-citrate complex.^[27]^ To authors’ knowledge, there are no studies regarding calcium citrate as an allergen.

Specific inhibitors of platelet function have been added to anticoagulants in attempts to minimize preanalytical activation in vitro. An example of this strategy comprises citrate, theophylline, adenosine, and CTAD. Theophylline and dipyridamole inhibit cAMP phosphodiesterase activity, and adenosine stimulates membrane adenylyl cyclase. The consequent increase in platelet cAMP and the inhibition of Ca^2+^-mediated responses lead to a reduction in platelet activation.

Few studies were performed to investigate the effect of anticoagulants on the platelet-rich plasma. Lei et al^[28]^ showed that ACD and CTAD were superior to heparin and citrate in maintaining the integrity of platelet structures and preventing the platelet spontaneous activation. ACD-PRP and CTAD-PRP released more TGF-beta1 and significantly enhanced the proliferation of human marrow stromal cells compared to heparin-PRP and citrate-PRP. In another thesis, Giraldo et al^[29]^ found that anticoagulants did not significantly influence cell counts or growth factor concentrations in equine PRP. However, the author stated that ACD-B was the worst anticoagulant evaluated.

## 4 Conclusion

Our case shows that safety of use of PRP is not absolutely sure. The pure autologous tissue is safe, but preparation for its use can substantially decrease this safety. In our patient, only limited skin reaction to calcium citrate was observed, but general reaction leading to anaphylactic shock cannot be excluded. In order to reduce the risk of side effects skin test should be performed but as there were no records of allergic diseases on family and patients medical history this should apply to all patients.

## Author contributions

**Conceptualization:** Michal Latalski.

**Data curation:** Michal Latalski, Anna Danielewicz, Marek Fatyga.

**Formal analysis:** Michal Latalski, Tomasz Szponder, Tomasz Bielecki.

**Investigation:** Michal Latalski, Alicja Walczyk, Anna Danielewicz.

**Methodology:** Michal Latalski, Marek Fatyga, Tomasz Szponder, Tomasz Bielecki.

**Validation:** Alicja Walczyk.

**Writing – original draft:** Michal Latalski, Tomasz Szponder, Tomasz Bielecki.

**Writing – review & editing:** Michal Latalski, Erich Rutz, Tomasz Bielecki.

Michal Latalski orcid: 0000-0002-7919-0294.
